# Effects of COVID-19 stress, proximity, and adverse childhood experiences on healthcare workers’ mental health

**DOI:** 10.3389/fpsyg.2023.1228515

**Published:** 2023-09-01

**Authors:** Tannaz Mirhosseini, Andrea D. Guastello, Lourdes P. Dale, Nicola Sambuco, Brandon R. Allen, Carol A. Mathews

**Affiliations:** ^1^Department of Psychiatry, College of Medicine, University of Florida, Gainesville, FL, United States; ^2^Department of Clinical and Health Psychology, College of Public Health and Health Professions, University of Florida, Gainesville, FL, United States; ^3^UF Center for OCD, Anxiety, and Related Disorders, University of Florida, Gainesville, FL, United States; ^4^Evelyn F. and William L. McKnight Brain Institute, University of Florida, Gainesville, FL, United States; ^5^Department of Psychiatry, College of Medicine-Jacksonville, University of Florida, Jacksonville, FL, United States; ^6^Department of Emergency Medicine, College of Medicine, University of Florida, Gainesville, FL, United States

**Keywords:** healthcare workers, COVID-19, stress, anxiety, depression, adverse childhood experiences, mental health, cross-sectional study

## Abstract

Past research has shown that healthcare workers (HCWs) experience high levels of psychological distress during epidemics and pandemics, resulting in cascading effects that have led to chronically understaffed hospitals and healthcare centers. Due to the nature of their responsibilities and workplace stress, HCWs are among vulnerable groups especially during global health crises. During COVID-19 many healthcare workers reported greater symptoms of anxiety, depression, and COVID-19 related worries. Furthermore, adverse childhood experiences increase vulnerability for psychological conditions, especially during pandemics. This study sets out to (1) investigate the moderating effects of adverse childhood experiences on healthcare workers’ COVID-19 related stressors and depression/anxiety symptoms, and (2) investigate the moderating effects of adverse childhood experiences on proximity to the COVID-19 virus and depression/anxiety symptoms. Participants included 438 employed HCWs recruited from academic medical centers and smaller healthcare agencies in northcentral Florida between October to December 2020. Mean age of participants was 38.23 (*SD* = 11.5) with most of the HCWs being white (72.1%), non-Hispanic (86.8%) and female (82%). Healthcare workers completed several online questionnaires, including the Adverse Childhood Experiences scale, Patient Health Questionnaire, Generalized Anxiety Disorder Scale, a COVID-19 specific worries scale, and a Social Proximity to COVID-19 scale. Healthcare workers experiencing specific COVID-19 worries reported experiencing anxiety and depressive symptoms. A significant positive interaction was seen between childhood adverse experiences globally and COVID-19 worries on anxiety symptoms. A significant positive interaction was observed between childhood maltreatment specifically and COVID-19 worries on depressive symptoms. Additionally, a positive interaction effect was seen between childhood adverse experiences and COVID-19 social proximity for both depression symptoms and anxiety symptoms. Findings from the present study indicate that adverse childhood experiences strengthen the relationship between COVID-19 worry/proximity and negative psychological symptoms. Vulnerable populations such as individuals who have experienced ACEs could benefit from targeted and specific interventions to cope with the collective trauma experienced globally due to COVID-19. As COVID-19 becomes endemic, hospital leadership and authorities should continue addressing COVID-19 worries and HCWs’ psychological symptoms through mental health support and organizational interventions.

## 1. Introduction

The onset of the COVID-19 pandemic caused many individuals to experience specific COVID-19 related worries, such as infecting family members with COVID-19, or becoming seriously ill from COVID-19 (Hidaka et al., 2021). These worries were related to greater anxiety and depressive symptoms more generally (Gupta et al., 2021). The healthcare workforce was particularly at risk for developing COVID-19 related worries as well as mental health symptoms, resulting in cascading effects that have led to chronically understaffed hospitals and healthcare centers (Gupta et al., 2021; Søvold et al., 2021). Understanding the risk factors that make individuals more vulnerable to pandemic related worries and associated mental health symptoms is an important public health concern. Prior to the pandemic, adverse childhood experiences were shown to predict mental health disorders in adulthood (McKay et al., 2022; Tzouvara et al., 2023). However, little is known about the interaction effect between adverse childhood experiences and COVID-19 specific worries on healthcare workers’ (HCWs) psychological symptoms. Similarly, the interaction effect between adverse childhood experiences and COVID-19 social proximity on HCWs’ psychological symptoms has also not been explored. COVID-19 social proximity refers to the impact COVID-19 has had on an individual’s social circle.

In the general population, the COVID-19 pandemic has negatively impacted individuals’ mental well-being and coincided with increased levels of anxiety, depression, and posttraumatic stress symptoms (Prout et al., 2020; Marvaldi et al., 2021). Recent research has shown that these adverse mental health outcomes have been exacerbated in HCWs, potentially due to their proximity to COVID-19 patients, COVID-19 quarantine rules and those rules changing, and work environment (Labrague and Santos, 2020; Dobson et al., 2021; Tiete et al., 2021). Similar trends have been seen in non-clinical staff who work in healthcare settings, such as custodians and technicians (Dobson et al., 2021; Jang et al., 2021). A meta-analysis identified 38 studies that reported an increase in mental health symptoms among doctors, nurses, and allied health workers since the start of the pandemic (Saragih et al., 2021), with a pooled prevalence among these HCWs for anxiety of 40, and 37% for depression.

Psychological symptoms have been seen in the United States in both general and healthcare populations (Amsalem et al., 2021; Guastello et al., 2022). Additionally, studies have shown that nurses in particular are at higher vulnerability for psychological distress during infectious disease outbreaks (Greenberg et al., 2020; Magalhaes et al., 2021). A five-month longitudinal study of HCWs in the United States in the early stages of the COVID-19 pandemic found that anxiety and depression symptoms were related to decreased fulfilment and elevated burnout (Guastello et al., 2022).

The burden of the COVID-19 pandemic has had major impacts on HCWs and the healthcare system. This burden has impacted the physical and mental health of individuals who work in healthcare (Luo et al., 2020). At the onset of COVID-19 certain worries were prevalent such as worrying about the health of family and friends, due to the possibility of bringing the virus home, as well as an individual’s own health (Hidaka et al., 2021). These COVID-19 specific worries can in turn increase other anxiety and depressive symptoms. For example, higher perceived risk has been shown to predict higher depressive symptoms (Kim et al., 2022). In particular, this is seen in HCWs due to their proximity to the illness and the uncertainty involved with medical care and precautions needed. Additionally, pandemics elicit anticipatory anxiety for both real and perceived threats which can create high stress environments in hospitals and burnout in HCWs (Denning et al., 2021). Similarly, Carmassi and colleagues found that HCWs were at a higher risk for PTSD during pandemics due to infection rates, high mortality, and the constant change of guidelines experienced during COVID-19 (Carmassi et al., 2020).

In HCWs, anxiety is commonly seen during pandemics due to the uncertainty and anticipation that comes from an unknown disease (Labrague and Santos, 2020). COVID-19 specific worries in this population can be due to fear of being infected, fear of unknowingly infecting others, lack of personal protective equipment, and lack of access to testing (Mo et al., 2020; Shanafelt et al., 2020).

Kim and colleagues found that higher perceived COVID-19 risk predicted greater depression symptoms among the general population in South Africa (Kim et al., 2022). A U.S. sample of young adults ages 18 to 25 indicated that this population reported up to a 55% increase in COVID-19 related stressors (Ballou et al., 2020). Importantly, past research has shown that among young adults COVID-19 related worry is related to negative mental health outcomes (Rogers et al., 2020; Mayorga et al., 2022). Research has also shown that proximity to an individual with the SARS-COV2 virus that causes COVID-19 can also have a psychological impact on the people around them (Su et al., 2020). Several studies have reported on the connection between social proximity to COVID-19 cases and increased anxiety and depression (Su et al., 2020; Wang et al., 2020; Shabahang et al., 2021; Vigo et al., 2021).

Another factor that can have a negative impact on mental health outcomes is adverse childhood experiences (ACEs). ACEs are identified as experiences that can negatively harm a child (0–17 years old). These experiences can be in the form of childhood maltreatment (emotional, physical, and sexual abuse), neglect (emotional and physical), familial challenges and dysfunction (caregiver separation, poor or impaired caregiver mental health, and caregiver drug abuse) (Felitti et al., 1998; LeMoult et al., 2020). It is estimated that 61% of US adults have experienced at least one ACE, and approximately 16% have experienced four or more categories (Merrick et al., 2019). These experiences are associated with an increase in psychiatric disorders (such as depression and anxiety) in adolescence and adulthood (Felitti et al., 1998; Cicchetti and Toth, 2005; Cicchetti, 2016; Iob et al., 2022). For example, a study of 1,142 participants aged 22–24 in the Chicago Longitudinal Study found that ACEs scores were related to increased depressive symptoms in early adulthood (Mersky et al., 2013). These relationships have also been examined by some research teams during the pandemic. A cross-sectional survey of 1,399 German adults found that ACEs were a significant risk factor for an increase in depression during the first wave of the pandemic (Clemens et al., 2022). ACEs put individuals at a higher vulnerability for psychological conditions, and several studies have examined the relationship between ACEs and negative mental health symptoms during the pandemic (Castellini et al., 2022; Kim et al., 2022; Békés et al., 2023). However, this specific relationship has not been extensively examined with HCWs’ to the others knowledge at the time of the study.

HCWs’ mental health symptoms during pandemic times is well-documented (Labrague and Santos, 2020; Amsalem et al., 2021; Saragih et al., 2021; Guastello et al., 2022). However, less is known about the way past adverse childhood experiences can impact their worries about the pandemic and/or their own proximity to COVID-19 and their mental health and well-being. The current study aims to examine the relationships between these variables in HCWs during an early stage of the COVID-19 pandemic (from October to December 2020). The aims of this study were:

Aim 1: To examine the role that adverse childhood experiences plays in the relationship between COVID-19 specific worries and negative psychological symptoms.

*Hypothesis 1.1*: We hypothesized an interaction between COVID-19 specific worries and adverse childhood experiences, such that the association between HCWs COVID-19 specific worries and depression/anxiety would be stronger in participants who had more adverse childhood experiences.

Aim 2: To examine the role that adverse childhood experiences plays in the relationship between proximity to COVID-19 and negative psychological symptoms.

*Hypothesis 2.1*: We hypothesized an interaction between proximity to COVID-19 cases and adverse childhood experiences, such that the association between HCWs proximity to COVID-19 cases and depression/anxiety would be stronger in participants who had more adverse childhood experiences.

## 2. Methods

### 2.1. Procedures

All procedures in this study were approved by the University of Florida’s Institutional Review Board. Participants were largely recruited from two academic medical centers in north central Florida. Announcements were posted throughout clinics, hospitals, and nursing homes in Florida via brochures emailed to relevant departments or clinical services from an administrator. Also, flyers were given to smaller medical groups and private practices in two cities near the academic medical centers. Additionally, the study was incorporated into the comprehensive Healthcare Worker Exposure Responses & Outcomes (HERO) registry of studies (heroesrearch.org). In order to maintain anonymity, the exact location of a participant’s workplace was not included in their responses. Using a QR code or link provided on the brochure, participants were directed to Research Electronic Data Capture (REDCap), a secure survey service, where their responses to the survey questions were recorded. This study is part of a larger dataset which had a 5-month longitudinal data collection (Guastello et al., 2022.) Recruitment was on a rolling basis between October and December 2020. The present study focused on solely baseline findings. Lastly, participants were reimbursed with $10 Amazon gift cards for completing the baseline surveys.

### 2.2. Participants

Participants included 438 employed HCWs recruited from academic medical centers and smaller healthcare agencies in north central Florida. Specific workplace location was not collected to protect the participants anonymity, however based on the collection of the zip code data, the majority of the participants resided in Florida (95.3%). Mean age of participants was 38.23 (*SD* = 11.5) with most of the HCWs being white (72.1%), non-Hispanic (86.8%) and female (82%). See Table 1 for a summary of the sample characteristics.

**Table 1:** Healthcare workers demographic.

	*n*	%
*Sex*		
Female	357	81.5
Male	73	16.7
Missing	8	1.8
*Racial groups*		
American Indian	3	0.7
Asian	27	6.2
Black/African American	58	13.2
Multiracial	17	3.9
Native Hawaiian	1	0.2
White	316	72.1
Other	16	3.7
Missing	7	1.6
*Ethnicity*		
Non-Hispanic	380	86.8
Hispanic/Latinx	46	10.6
Missing	12	2.7

### 2.3. Measures

#### 2.3.1. Adverse childhood experiences

The Adverse and Traumatic Experiences Scale (Dale et al., 2020, 2022; Kolacz et al., 2020) was administered to assess adverse childhood experiences (ACEs). This is a 30-question instrument that asks about adversity and traumatic experiences, and covers both childhood and adulthood. This measure is comprised of items from: ACES (Felitti et al., 1998), Trauma History Questionnaire (Hooper et al., 2011), Life Events Checklist for DSM-5 (Weathers et al., 2013), and Brief Trauma Questionnaire (Schnurr et al., 1999). The participants specify the occurrence and impact of each event via a 5-point Likert scale (0 = *event did not occur*, 1 = *occurred and had no impact on my life*, 2 = *minimal impact on my life*, 3 = *some impact on my life*, and 4 = *big impact on my life*). The items are grouped to form the following scales: Childhood Adverse Experiences, Childhood Maltreatment, Intimate Partner Maltreatment, Other Person Maltreatment, Life-threatening Situations, Sudden Losses, and Personal Health Situations. For the purpose of examining ACEs, the subscales Childhood Adverse Experiences and Childhood Maltreatment were used in this study. The subscale Childhood Adverse Experiences captures caregiver unavailability, caregiver separation, caregiver drug abuse, caregiver medical or mental illness, caregiver experience of emotional/physical/sexual abuse. The subscale Childhood Maltreatment captures emotional abuse, physical assault, sexual abuse of the participant by the caregiver. In our sample, the internal consistency for Childhood Adverse Experiences was 0.72, and for Childhood Maltreatment it was 0.69.

#### 2.3.2. Depression

The Patient Health Questionnaire (PHQ-8; Kroenke et al., 2001) was administered to assess depression symptoms. On the PHQ-8 the participant is asked to rate over the last two weeks how frequently they have experienced the following symptoms of depression: low mood, anhedonia, hyper/hyposomnia, increased/decreased appetite, difficulty concentrating, self-blame, psychomotor retardation/agitation. Responses are recorded on a 4-point Likert scale ranging from 0 (*not at all*) to 3 (*nearly every day*). The total score of the eight items of the PHQ-8 ranges from 0 to 24, with a cut point of 10 indicating clinically significant symptoms of depression (Kroenke et al., 2009). In our sample, the internal consistency of the PHQ-8 was 0.89.

#### 2.3.3. Anxiety

The Generalized Anxiety Disorder Scale (GAD-7; Spitzer et al., 2006) was administered to assess anxiety symptoms. On the GAD-7 the participant is asked to rate how frequently over the last two weeks they have experienced the following symptoms of anxiety: feeling nervous, anxious or on edge; difficulty controlling worry; psychomotor agitation; trouble relaxing; general worries; fear that something terrible will happen; and irritability. Responses are recorded on a 4-point Likert scale ranging from 0 (*not at all*) to 3 (*nearly every day*). The total score of the 7 items of the GAD-7 ranges from 0 to 21, with scores exceeding 10 representing clinically significant anxiety. In our sample, the internal consistency of the GAD-7 was 0.93.

#### 2.3.4. COVID-19 specific worries

A COVID-19 specific worries and experiences questionnaire was created by the research team which consisted of various HCWs including psychiatrists, psychologists, and emergency medicine physicians. The scale consisted of 13 items, rated on a 0 to 3 scale with the following descriptors: 0 (Not worried), 1 (A little worried), 2 (Somewhat worried), and 3 (Very worried). The principal components analysis produced 3 terms accounting for 68.12% of the variance. The items and their accompanying factor loadings are presented in Table 2, and the derivation of the subscales can be found in the paper (Guastello et al., 2022). The first component, termed Infection Worries, was comprised of seven items regarding worry about self and family members being infected and/or becoming seriously ill from COVID-19; the internal consistency of this factor was 0.89. The second component, termed Childcare Worries, was comprised of three items related to worries about emotional wellbeing, education, and behavior; the internal consistency of this factor was 0.77. Lastly, the third component, termed Economic Worry, was comprised of three items regarding financial concerns such as accessing or paying for childcare, loss of job, and having trouble paying bills; the internal consistency of this factor was 0.86. See Table 2 for full factor analysis. The Infection Worry factor, also referred to as the COVID-19 Worries subscale, was utilized in this study.

**Table 2:** Principal components analysis with varimax rotation for COVID-19 specific worries.

Item	Infection worries	Childcare worries	Economic worries
*How worried are you that you will…*			
Infect an immediate family member if you get COVID-19	0.83*	0.04	0.10
Be infected with COVID-19 in your home or community (e.g., while at grocery store or pharmacy)	0.73*	0.14	0.05
Become seriously ill because of COVID-19	0.83*	−0.00	0.12
Be infected with COVID-19 while providing medical care	0.68*	0.20	0.13
*How worried are you that an immediate family member…*			
Will be infected with COVID-19	0.82*	0.13	0.04
Will become seriously ill with COVID-19	0.72*	0.27	0.20
Is having trouble coping with fear of getting COVID-19	0.47*	0.44	−0.01
*How worried are you about the following…*			
My child’s emotional wellbeing	0.33	0.76*	0.22
My child’s education	0.11	0.83*	0.20
My child’s behavior at home	0.05	0.74*	0.21
*How worried are you that you will…*			
Accessing or paying for childcare	0.06	0.21	0.75*
Lose your job	0.19	0.21	0.86*
Have trouble paying your bills	0.18	0.21	0.75*

Numbers indicate factor loadings.

* Items included in the indicated factor.

#### 2.3.5. Social proximity to COVID-19

A Social Proximity to COVID-19 scale was created by the research team to measure the degree to which COVID-19 was impacting an individual’s social circle (Table 3). The scale originally consisted of 8 items, though one item (i.e., a member of my household passed away from COVID-19) was removed due to low endorsement. Each item was rated on a 0 to 4 scale with the following descriptors: 0 (*Did not occur*), 1 (*Occurred, and no impact on my life*), 2 (*Minimal impact on my life*), 3 (*Some impact on my life*), 4 (*Big impact on my life*). A principal components analysis for the remaining 7 items was conducted using varimax rotation. Items were considered to load on a factor if they had a factor loading of ≥0.50. Items that loaded onto more than one factor were included in all factors. The principal components analysis produced 3 factors that accounted for 72.65% of the variance. The factor loadings are presented in Table 3. The first component, termed Known Infections, contained three items regarding the social proximity to COVID-19 infections; internal consistency was 0.74. The second component, termed Household Infections, contained two items regarding members of the participant’s household and they themselves being infected; internal consistency was 0.69. The third component, termed, Deaths, contained two items regarding social proximity to COVID-19 related deaths; internal consistency 0.53. See Table 3 for full factor analysis.

**Table 3:** Principal components analysis with varimax rotation for social proximity to COVID-19.

Item	Known infections	Household infections	Deaths
I personally know someone (not close friend or relative) diagnosed with COVID-19	0.88*	0.04	0.15
I know someone at work that was diagnosed with COVID-19	0.85*	0.02	−0.04
A close friend or relative was diagnosed with COVID-19	0.60*	0.31	0.34
I cared for a member of my household that was diagnosed with COVID-19	0.08	0.86*	0.13
I have been diagnosed with COVID-19	0.06	0.86*	−0.05
A close friend or relative passed away from COVID-19	−0.02	0.05	0.91*
I personally know someone (not close friend or relative) who passed away from COVID-19	0.47	0.01	0.64*

* Items included in the indicated factor.

### 2.4. Overview of statistical analyses

All analyses were conducted in SPSS Statistics Version 26 [IBM Corp (SPSS Inc.), 2019], using data collected at baseline. Descriptive statistics were first used to assess the distributions, normality, missing data, and any outliers among variables. Out of 438 participants, only 177 had children and answered for the variable Childcare Worries. Therefore, this subscale was dropped due to the significant loss in sample size. Additionally, the subscale Economic worries was not used due to high cross-loading with Childcare Worries subscale. For Aims 1 and 2, four separate hierarchical linear regressions were conducted in order to examine the models with COVID-19 worries, COVID-19 social proximity, childhood adverse experiences and childhood maltreatment as predictors, and depression and anxiety as outcome variables. An interaction variable was computed for the variables of interest and added to the second block of the regression.

## 3. Results

### 3.1. Descriptive statistics and preliminary findings

The mean number of childhood adverse experiences reported in the sample was 6.35 (*SD* = 5.8), and the mean number of childhood maltreatment experiences reported in the sample was 2.11 (*SD* = 2.9). A floor effect was observed for both scales, with 21.9% of HCWs reporting no childhood adverse experiences, and 54.1% reporting no childhood maltreatment. Variability was seen across all variables. See Table 4 for a summary of the findings. Correlational analyses were used to examine the relationship between the variables of interest. See Table 5 for a summary of the findings.

**Table 4:** Descriptive statistics for predictor and outcome variables.

	*n*	M(SD)	Median	Range
Childhood adverse experiences	430	6.34 (5.8)	5	0–24
Childhood maltreatment	431	2.11 (2.9)	0	0–12
COVID-19 specific worries	435	10.72 (5.3)	11	0–21
Childcare worries	177	4.75 (3.41)	4	0–12
Economic worries	435	1.98 (2.23)	1	0–10
COVID-19 known infections	432	5.47 (3.1)	5	0–12
Household infections	435	0.71 (2.15)	0.00	0–20
COVID-19 deaths	435	1.31 (1.87)	0.00	0–8
Depression	434	6.08 (5.3)	5	0–24
Anxiety	434	5.55 (5.4)	4	0–21

Total sample was 438 participants.

**Table 5:** Correlations.

	M(SD)	(1)	(2)	(3)	(4)	(5)	(6)	(7)	(8)	(9)
1. Childhood adversity	6.34 (5.8)	–								
2. Childhood maltreatment	2.11(5.9)	0.62**	–							
3. COVID worry	10.72 (5.3)	0.10*	0.07	–						
4. Childcare worry	4.75 (3.41)	0.26**	0.24**	0.41**	–					
5. Economic worry	1.98 (2.23)	0.23**	0.22**	0.24**	0.51**	–				
6. Known infection	5.47 (3.1)	0.09	0.01	0.31**	0.36**	0.16**	–			
7. Household infection	0.71 (2.15)	0.07	0.05	−0.22**	0.02	0.15**	0.23**	–		
8. COVID-19 deaths	1.31 (1.87)	0.00	−0.01	0.13**	0.11	0.09	0.42**	0.07	–	
9. Depression symptoms	6.08 (5.3)	0.32**	0.33**	0.30**	0.44**	0.39**	0.17**	0.05	0.06	–
10. Anxiety symptoms	5.55 (5.4)	0.28**	0.31**	0.27**	0.38**	0.34**	0.13**	0.03	0.03	0.77**

*p ≤ 0.05, **p ≤ 0.01.

### 3.2. Aim 1

**Figure 1:**
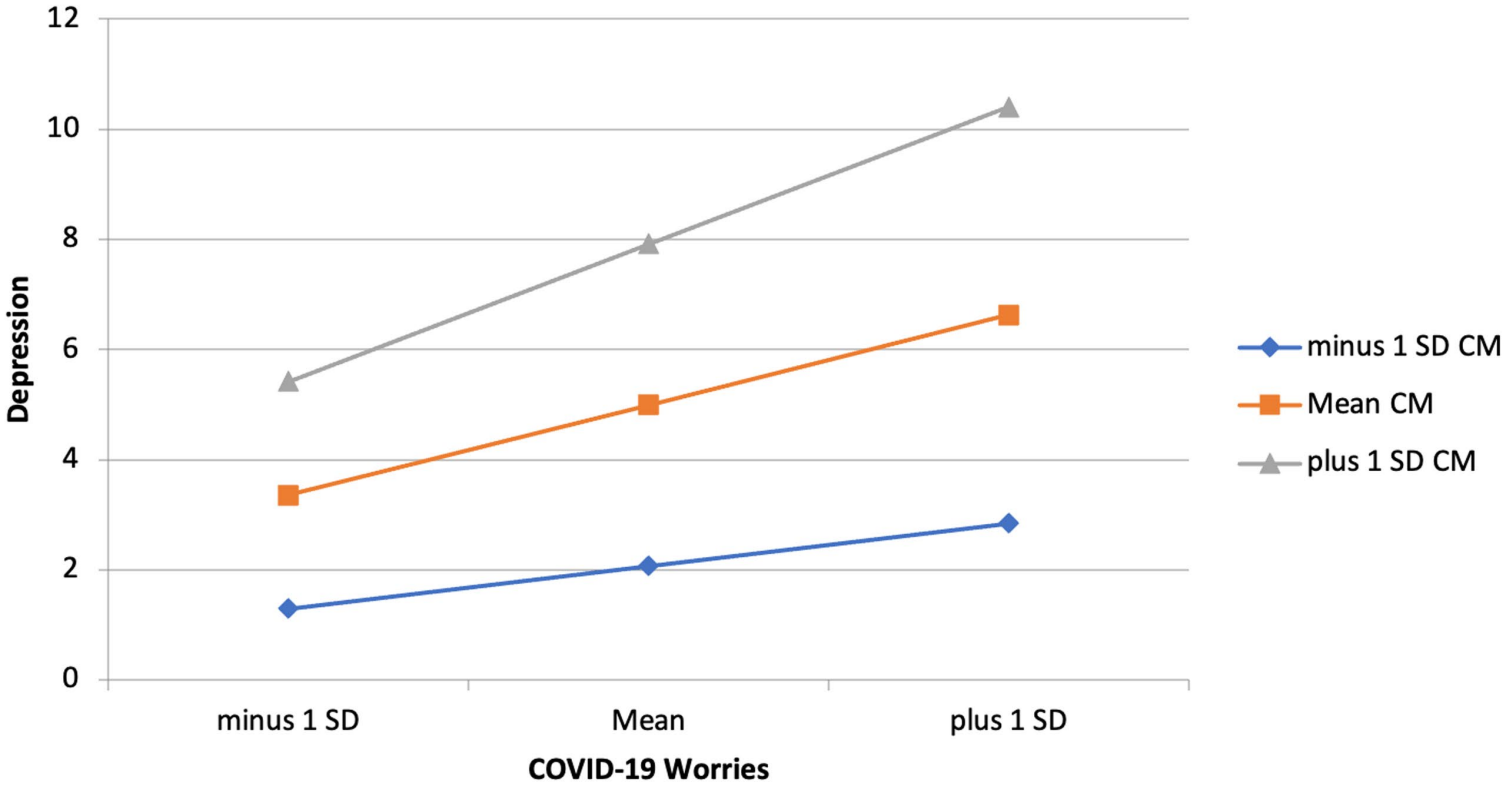
Interaction effect of childhood maltreatment and COVID-19 worries on depression.

To investigate the effects of childhood adverse experiences and childhood maltreatment on the relationship between COVID-19 related stressors and depression, main effects were examined in step one of the linear regressions. Model 1 included childhood adverse events, childhood maltreatment, and COVID-19 worries as predictors and depression as the outcome, shows that COVID worry significantly predicted depression (*B* = 0.27, *p* < 0.001), childhood adverse experiences significantly predicted depression (*B* = 0.14, *p* = 0.005), and childhood maltreatment significantly predicted depression (*B* = 0.40, *p* < 0.001). Model 2 included all of these predictors and interaction terms between COVID-19 worry and the two childhood experiences variables. The *R*^2^ change between model 1 and model 2 was 0.29, with a significant *F* change (*F* = 132.01, *p* < 0.001). Model 1 explained 20% of the variance, whereas model 2 explained 49% of the variance, indicating that the model fit improved when accounting for the interactions between childhood maltreatment and childhood adverse experiences with COVID worry. The relationship between COVID worry and depression was stronger among individuals who scored higher in childhood maltreatment (*B* = 0.06, *p* = 0.01). The same interaction was not seen for childhood adverse experiences, suggesting that the level of exposure to childhood adverse experiences did not influence the relationship between COVID worries and depression. See Table 6 for hierarchical linear regression analyses, and Figure 1 for graphical representation of the interaction.

**Table 6:** Hierarchical linear regression of COVID-19 worry on depression.

Model		Unstandardized coefficients	Standardized coefficients		Sig.	*R*^2^ change	Cumulative *R*^2^
	B	*SE*	Beta
1	(Constant)	1.52	0.56		0.007	–	0.21
COVID Worry	0.27	0.04	0.27	<0.001			CAE	0.14	0.05	0.16	0.005			CM	0.40	0.10	0.22	<0.001		
2	(Constant)	0.83	0.45		0.063	0.29	0.50
COVID Worry	0.19	0.04	0.19	<0.001			CAE	0.10	0.04	0.11	0.016			CM	0.41	0.08	0.23	<0.001			Worry X CAE	0.02	0.02	0.16	0.321			Worry X CM	0.06	0.02	0.40	0.012		

Dependent variable is depression as assessed by PHQ-8 total score. CAE, Childhood Adverse Experiences; CM, Childhood Maltreatment; COVID Worry, COVID-19 specific worries.

**Figure 2:**
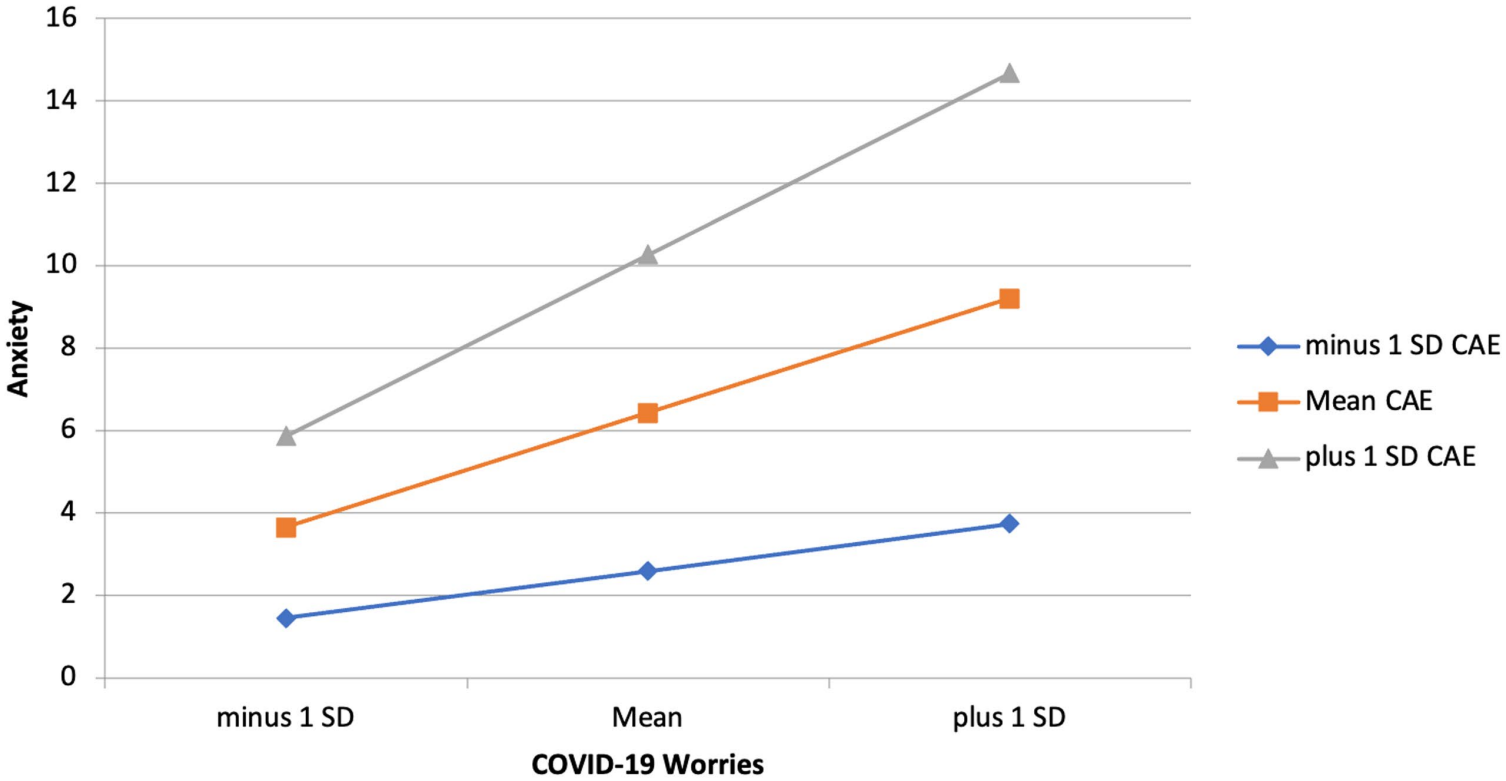
Interaction effect of childhood adverse experiences and COVID-19 worries on anxiety.

To investigate the effects of childhood adverse experiences and childhood maltreatment on the relationship between COVID-19 related worries and anxiety main effects were examined. Model 1 shows that COVID worry significantly predicts anxiety (*B* = 0.25, *p* < 0.001), and childhood maltreatment significantly predicts anxiety (*B* = 0.43, *p* < 0.001). The *R*^2^ change between model 1 and model 2 was 0.34, with a significant *F* change (*F* = 128.01, *p* < 0.001). Model one consisting of main effects explains 17% of the variance, whereas model 2 with interactions added explains 50% of the variance. This indicates that the regression model improved when accounting for the interactions between childhood maltreatment and childhood adverse experiences with COVID worry. The relationship between COVID worry and anxiety was stronger among individuals who scored higher in childhood adverse experiences (*B* = 0.05, *p* = 0.03). The same interaction was not seen for childhood maltreatment, meaning that the level of exposure to childhood maltreatment did not influence the relationship between COVID worries and anxiety. See Tables 3, 4 for hierarchical linear regression analyses, and Figure 2 for graphical representation of the interaction.

### 3.3. Aim 2

**Figure 3:**
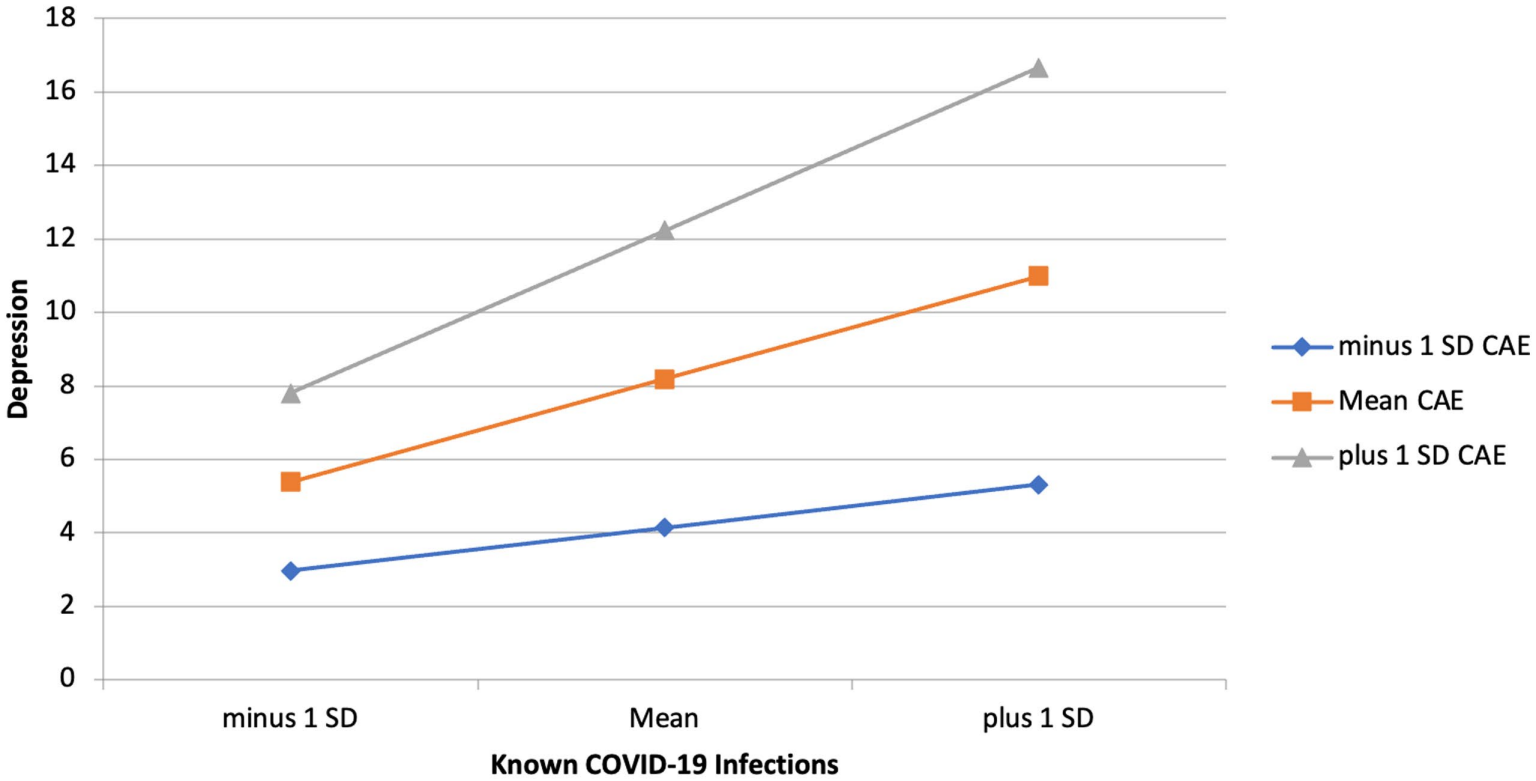
Interaction effect of childhood adverse experiences and known COVID-19 infections on depression.

To investigate the effects of childhood adverse experiences and childhood maltreatment on the relationship between Known COVID-19 Infection and depression main effects were examined. Model 1 shows that Known COVID worry significantly predicted depression (*B* = 0.26, *p* = 0.003), childhood adverse experiences significantly predicted depression (*B* = 0.15, *p* = 0.005) and childhood maltreatment significantly predicted depression (*B* = 0.41, *p* < 0.001). Household infections and COVID-19 Deaths did not significantly predict depression and therefore were not included in the subsequent interaction models. The *R*^2^ change between model 1 and model 2 was 0.31, with a significant *F* change (*F* = 122.00, *p* < 0.001). Model one consisting of main effects explained 16% of the variance, whereas model 2 with interactions added explained 47% of the variance. This indicates that the regression model improved when accounting for the interactions between childhood maltreatment and childhood adverse experiences with Known COVID-19. The relationship between Known COVID-19 infection and depression was stronger among individuals who scored higher in childhood adverse experiences (*B* = 0.05, *p* = 0.03). The same interaction was not seen for childhood maltreatment, meaning that the level of exposure to childhood maltreatment did not influence the relationship between Known COVID-19 infection and depression. See Tables 3–5 for hierarchical linear regression analyses, and Figure 3 for graphical representation of the interaction.

**Figure 4:**
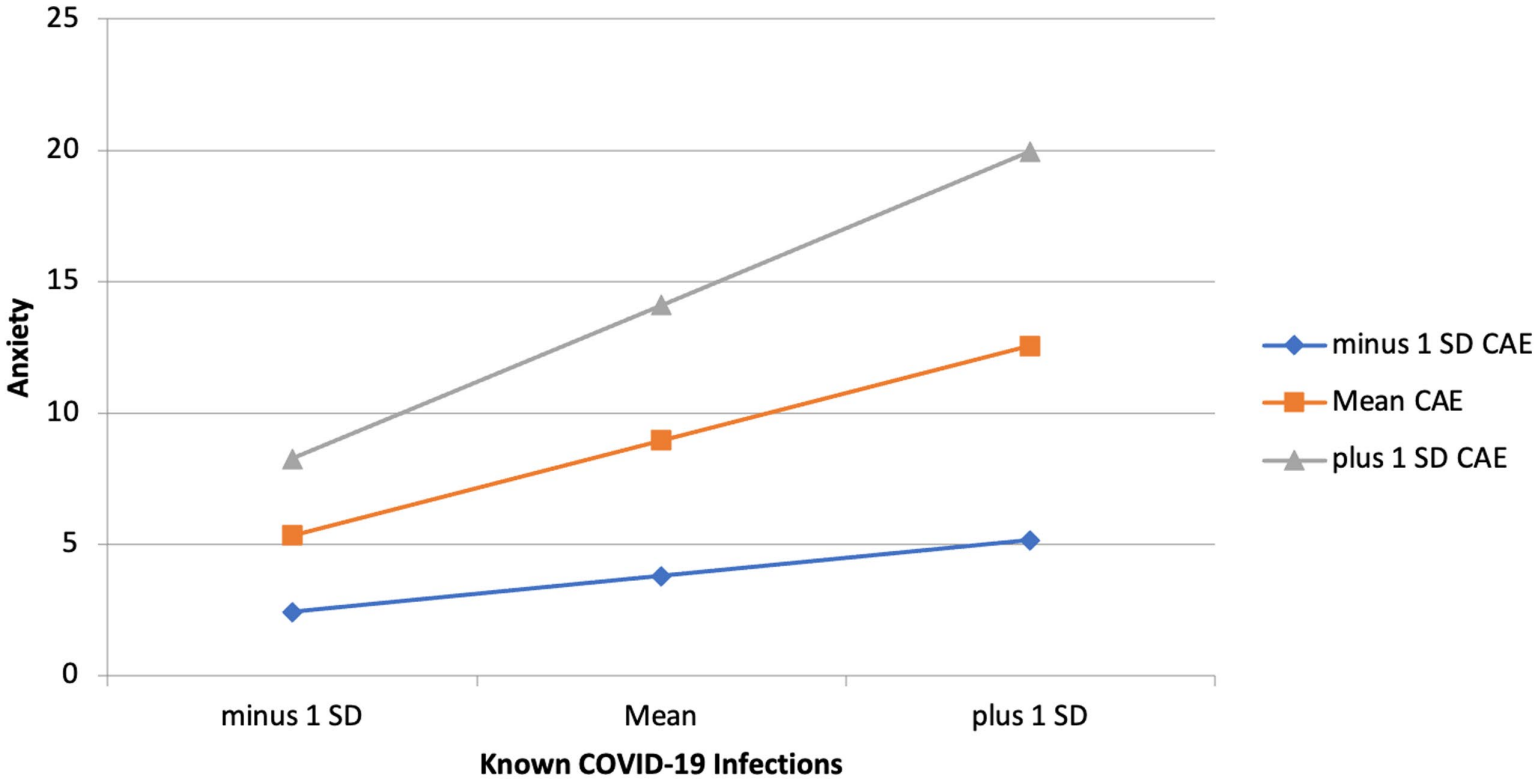
Interaction effect of childhood adverse experiences and known COVID-19 infections on anxiety.

To investigate the effects of childhood adverse experiences and childhood maltreatment on the relationship between Known COVID-19 Infection and anxiety main effects were examined. Model 1 shows that Known COVID Infection significantly predicts anxiety (*B* = 0.21, *p* = 0.024), childhood adverse experiences on anxiety is significant (*B* = 0.11, *p* = 0.046), and childhood maltreatment on anxiety is significant (*B* = 0.43, *p* < 0.001). Household infections and COVID-19 Deaths did not significantly predict depression and therefore were not included in the interactions. The *R*^2^ change between model 1 and model 2 was 0.37, with a significant *F* change (*F* = 149.04, *p* < 0.001). Model one consisting of main effects explains 12% of the variance, whereas model 2 with interactions added explains 49% of the variance. This indicates that the regression model improved when accounting for the interactions between childhood maltreatment and childhood adverse experiences with Known COVID. The relationship between Known COVID-19 infection and anxiety was stronger among individuals who scored higher in childhood adverse experiences (*B* = 0.07, *p* = 0.003). The same interaction was not seen for childhood maltreatment, meaning that the level of exposure to childhood maltreatment did not influence did not influence the relationship between Known COVID-19 infection and anxiety. See Tables 3–6 for hierarchical linear regression analyses, and Figure 4 for graphical representation of the interaction.

## 4. Discussion

The current study aimed to examine the relationships between adverse childhood experiences, worries about the COVID-19 pandemic, proximity to COVID-19, and the mental health symptoms of HCWs during the early stage of the pandemic. The findings of this study can provide insights into the complex interplay between these variables and highlight the potential impact of childhood experiences on HCWs’ psychological well-being. Consistent with previous research documenting the mental health challenges faced by HCWs during the pandemic (Labrague and Santos, 2020; Amsalem et al., 2021; Saragih et al., 2021; Guastello et al., 2022), the study findings show a significant association between COVID-19 worries and negative psychological symptoms, including depression and anxiety. These results highlight the substantial burden placed on HCWs due to the pandemic-related concerns they face, emphasizing the importance of addressing these worries to support their well-being (Tables 7–9).

**Table 7:** Hierarchical linear regression of COVID-19 worry on anxiety.

Model	Unstandardized Coefficients		Standardized Coefficients	Sig.	*R*^2^ change	Cumulative *R*^2^	*B*	*SE*	Beta
1	(Constant)	1.37	0.58		0.019	–	0.17	COVID Worry	0.25	0.05	0.29	<0.001			CAE	0.10	0.05	0.11	0.059			CM	0.43	0.10	0.23	<0.001		
2	(Constant)	0.24	0.45		0.592	0.34	0.51	COVID Worry	0.19	0.04	0.18	<0.001			CAE	0.09	0.04	0.10	0.023			CM	0.29	0.08	0.19	<0.001			WorryXCAE	0.05	0.02	0.37	0.028			WorryXCM	0.03	0.02	0.23	0.160		

Dependent Variable is Anxiety as assessed by GAD-7 total score. CAE, Childhood Adverse Experiences; CM, Childhood Maltreatment; COVID Worry, COVID-19 specific worries.

**Table 8:** Hierarchical linear regression of social Proximity to COVID-19 on depression.

Model	Unstandardized Coefficients		Standardized Coefficients	Sig.	*R*^2^ change	Cumulative *R*^2^	*B*	*SE*	Beta
1	(Constant)	2.88	0.53		<0.001	–	0.16	Known COVID	0.26	0.09	0.15	0.003			Household	0.20	0.15	0.06	0.184			Death	−0.03	0.14	−0.01	0.813			CAE	0.15	0.05	0.16	0.005			CM	0.41	0.10	0.23	<0.001		
2	(Constant)	1.69	0.43		<0.001	0.31	0.47	Known COVID	0.19	0.07	0.11	0.006			Household	0.11	0.18	0.04	0.337			Death	−0.08	0.11	−0.03	0.464			CAE	0.13	0.04	0.14	0.002			CM	0.36	0.09	0.20	<0.001			KnownXCAE	0.05	0.02	0.38	0.025			KnownXCM	0.03	0.02	0.18	0.286		

Dependent variable is depression as assessed by PHQ-8 total score. Known COVID, Known COVID-19 infections; Household, COVID-19 Household Infections; Death, COVID-19 Deaths; CAE, Childhood Adverse Experiences; CM, Childhood Maltreatment.

**Table 9:** Hierarchical linear regression of social proximity to COVID-19 on anxiety.

Model	Unstandardized Coefficients		Standardized Coefficients	Sig.	*R*^2^ change	Cumulative *R*^2^	*B*	*SE*	Beta
1	(Constant)	2.89	0.56		<0.001	–	0.12	Known COVID	0.21	0.09	0.12	0.024			Household	0.10	0.15	0.03	0.517			Death	−0.04	0.15	−0.01	0.779			CAE	0.11	0.06	0.12	0.046			CM	0.43	0.11	0.24	<0.001		
2	(Constant)	0.99	0.44		0.025	0.37	0.49	Known COVID	0.22	0.07	0.11	0.001			Household	0.04	0.12	0.01	0.744			Death	−0.09	0.11	−0.03	0.445			CAE	0.11	0.04	0.12	0.010			CM	0.27	0.09	0.15	0.001			KnownXCAE	0.07	0.02	0.52	0.003			KnownXCM	0.01	0.03	0.10	0.589		

Dependent variable is anxiety as assessed by GAD-7 total score. Known COVID, Known COVID-19 infections; Household, COVID-19 Household Infections; Death, COVID-19 Deaths; CAE, Childhood Adverse Experiences; CM, Childhood Maltreatment.

One novel contribution of this study is the exploration of the role of adverse childhood experiences in moderating the relationship between COVID-19 worries and mental health outcomes among HCWs. The study hypotheses proposed that the association between COVID-19 worries, and depression/anxiety would be stronger for HCWs who had experienced more adverse childhood events. The results partially supported these hypotheses, as the interaction between childhood maltreatment and COVID-19 worries was found to significantly predict depression. Specifically, individuals with higher levels of childhood maltreatment demonstrated a stronger relationship between COVID-19 worries and depressive symptoms. Similar results have been seen in a study done in South Africa during the first wave of the COVID-19 pandemic which found that adults with histories of childhood trauma experience higher depressive impacts of perceived COVID-19 infection risk compared to individuals with no or minimal childhood trauma (Kim et al., 2022). These findings could imply that childhood maltreatment may amplify the impact of COVID-19 worries on HCWs’ mental health. Gaining insight into this interaction can inform interventions to support HCWs with a history of childhood maltreatment and alleviate the negative psychological consequences of COVID-19 worries.

Furthermore, the interaction between childhood adverse experiences and COVID-19 worries was found to significantly predict anxiety. This interaction was not seen with childhood maltreatment suggesting that individuals who have experienced childhood maltreatment will report anxiety independent of having COVID-19 specific worries. Similarly, to our findings, Békés and colleagues found that the number of reported adverse childhood experiences positively predicted reported levels of COVID-19 related fears, anxiety, and depression (2022). However, unlike our results, Castellini and colleagues found in a general population sample of 101 Italian women that individuals that experienced childhood trauma, specifically emotional abuse, had increased levels of distress at the onset of the pandemic compared to individuals that did not report emotional abuse (2022).

Moreover, the study examined the role of adverse childhood experiences in the relationship between HCWs’ proximity to COVID-19 cases and negative psychological symptoms. The hypothesis proposed that the association between proximity to COVID-19 cases and depression/anxiety would be stronger for HCWs with more adverse childhood experiences. When we examined the interaction effects, the relationship between Known COVID-19 infection and depression was stronger among individuals who scored higher in childhood adverse experiences and not childhood maltreatment. This finding builds on previous research done by Wang and colleagues which found that having knowledge of COVID-19 cases in relatives and family members was associated with elevated levels of depression symptoms (2020).

Similar interaction was seen when examining this model with anxiety symptoms as the outcome variable, results show that Known COVID-19 infection, childhood adverse experiences, and childhood maltreatments all significantly predicted anxiety symptoms. These results were hypothesized given the previous research on COVID-19 proximity and anxiety symptoms. In a housing compound in Guangzhou, China, the results of 403 participants showed that higher anxiety levels were associated with residents that knew of the presence of individuals with COVID-19 in their building (Su et al., 2020). Similarly, a web-based survey of 398 university students in Iran found that individuals who personally knew of someone with COVID-19 experienced more COVID-19 anxiety than individuals who did not (Shabahang et al., 2021). A study of university students in Vancouver found that knowledge of COVID-19 cases was associated with probability of elevated anxiety symptoms (Vigo et al., 2021). When examining the interaction effects for this model, the relationship between Known COVID-19 infection and anxiety was only stronger among individuals who scored higher in childhood adverse experiences and not childhood maltreatment.

The current study has several implications in line with current literature (Greenberg et al., 2020; Amsalem et al., 2021; Magalhaes et al., 2021), for understanding and addressing the mental health needs of HCWs during pandemic times. Firstly, it emphasizes the importance of recognizing and addressing the specific worries and concerns related to the COVID-19 pandemic that HCWs experience. Interventions and support systems should be implemented to help HCWs manage these worries effectively, providing them with resources and coping strategies to mitigate the negative psychological symptoms associated with the pandemic. Secondly, the study highlights the potential long-lasting impact of childhood maltreatment on HCWs’ mental health during the pandemic. Expanding on past literature, this study demonstrates how the presence of childhood adverse experiences such as caregiver unavailability, caregiver separation, caregiver drug abuse, caregiver medical or mental illness, caregiver experience of emotional/physical/sexual abuse can exacerbate the relationship between COVID-19 specific worries and anxiety, as well as proximity to COVID-19 (Known COVID-19 infection) and depression and anxiety.

Due to the nature of their responsibilities and workplace stress, HCWs are among vulnerable groups especially during global health crises. Considering the prevalence rates of ACEs in the United States, the results of this study emphasize the importance of keeping in mind HCWs who have experienced past aversive childhood experiences as these experiences can experience more psychiatric symptoms. In addition to daily life stressors, HCWs are responding to unknown virus outbreaks and patient crises. For them to provide effective care without experiencing burnout and significant depression and or anxiety, they need to attend to their own mental health well-being first. Seeking mental health care can be intimating and may be accompanied with stigma even in medical settings. Therefore, the sole presence of resources is not enough. Creating an environment where HCWs feel encouraged to seek out mental health services is imperative for hospital leaders, policymakers, and direct supervisors. Trauma-informed approaches and evidence-based interventions for individuals with a history of childhood maltreatment should be considered to enhance their resilience and well-being during these challenging times.

### 4.1. Strengths and limitations

One of the strengths of this study is the inclusion of non-clinical HCWs in the sample. Many studies on HCWs focus on doctors and nurses, and as prior research has shown non-clinical HCWs face psychological distress given the environment of their work (Dobson et al., 2021; Jang et al., 2021). Another strength of this study is the sample size which allowed for better statistical power to detect relationships. One of the limitations when examining this study is its limited diversity, the majority of the sample were white American women, and the sample was drawn from one health system in Florida. Gender of the participant could impact both adverse childhood experiences and the manifestation of psychological symptoms. Diversifying the data by examining multiple different academic medical centers with different policies will provide a more comprehensive insight to HCWs mental health given that it will provide a larger variance in gender, ethnicity, and socioeconomic status.

This data was collected from October to December 2020, a time where many COVID-19 outcomes were still uncertain. It is worth emphasizing that high-risk medical workers in these health systems gained access to COVID-19 vaccines starting from December 2020. The pandemic could have affected HCWs that participated in the first month of the study differently than HCWs that participated in the last month of the study given the availability of the vaccines and more understanding of the virus. Therefore, due to the rapid change in policies at both state level and hospital level, as well as the unpredictability and unknown nature of COVID-19 at the beginning stages of the pandemic, the generalizability of this data can be a limitation. Nevertheless, this study provides an insight into the impact COVID-19 had on HCWs at the end of the first year of the pandemic. Future research could explore the long-term impact of COVID-19 stress and adverse childhood experiences on HCWs’ mental health throughout different phases of the pandemic.

## 5. Conclusion

This study expands on previous literature regarding the impact of COVID-19 on HCWs’ mental health. The current study found that adverse childhood experiences strengthen the relationship between HCWs COVID-19 worries and proximity when predicting their psychological symptoms. As COVID-19 becomes endemic, hospital leaderships and authorities need to continue addressing COVID-19 worries and HCWs’ psychological symptoms through mental health support and organizational interventions. More specifically, vulnerable populations such as individuals who have ACEs will benefit from targeted and specific interventions to cope with the collective trauma experienced globally due to COVID-19.

## Data availability statement

The original contributions presented in the study are included in the article/supplementary materials, further inquiries can be directed to the corresponding author.

## Ethics statement

The studies involving human participants were reviewed and approved by the University of Florida Institutional Review Board. The patients/participants provided their written informed consent to participate in this study.

## Author contributions

TM conceptualized the manuscript, wrote the majority of the manuscript, and revised the manuscript. AG contributed funding for the parent study, conceptualized the manuscript, assisted with statistical analyses and write up, and revised the manuscript. LD conceptualized the parent study and inclusion of the measures of interest, and edited and revised the manuscript. NS conceptualized and secured funding for the parent study. BA and CM conceptualized the parent study and edited and revised the manuscript. CM contributed funding for the parent study. All authors contributed to the article and approved the final version.

## Funding

This work was supported in part by the University of Florida, Clinical and Translational Science Institute (supported in part by the NIH National Center for Advancing Translational Sciences under award number UL1TR001427), and in part by the Evelyn F. and William L. McKnight Brain Institute and the UF Center for OCD, Anxiety, and Related Disorders.

## Conflict of interest

The authors declare that the research was conducted in the absence of any commercial or financial relationships that could be construed as a potential conflict of interest.

## Publisher’s note

All claims expressed in this article are solely those of the authors and do not necessarily represent those of their affiliated organizations, or those of the publisher, the editors and the reviewers. Any product that may be evaluated in this article, or claim that may be made by its manufacturer, is not guaranteed or endorsed by the publisher.
